# Comparative genetic, proteomic and phosphoproteomic analysis of *C*. *elegans* embryos with a focus on *ham*-*1*/STOX and *pig*-*1*/MELK in dopaminergic neuron development

**DOI:** 10.1038/s41598-017-04375-4

**Published:** 2017-06-28

**Authors:** Sarah-Lena Offenburger, Dalila Bensaddek, Alejandro Brenes Murillo, Angus I. Lamond, Anton Gartner

**Affiliations:** Centre for Gene Regulation and Expression, School of Life Sciences, University of Dundee, Dundee, DD1 5EH UK

## Abstract

Asymmetric cell divisions are required for cellular diversity and defects can lead to altered daughter cell fates and numbers. In a genetic screen for *C*. *elegans* mutants with defects in dopaminergic head neuron specification or differentiation, we isolated a new allele of the transcription factor HAM-1 [HSN (Hermaphrodite-Specific Neurons) Abnormal Migration]. Loss of both HAM-1 and its target, the kinase PIG-1 [PAR-1(I)-like Gene], leads to abnormal dopaminergic head neuron numbers. We identified discrete genetic relationships between *ham*-*1*, *pig*-*1* and apoptosis pathway genes in dopaminergic head neurons. We used an unbiased, quantitative mass spectrometry-based proteomics approach to characterise direct and indirect protein targets and pathways that mediate the effects of PIG-1 kinase loss in *C*. *elegans* embryos. Proteins showing changes in either abundance, or phosphorylation levels, between wild-type and *pig*-*1* mutant embryos are predominantly connected with processes including cell cycle, asymmetric cell division, apoptosis and actomyosin-regulation. Several of these proteins play important roles in *C*. *elegans* development. Our data provide an in-depth characterisation of the *C*. *elegans* wild-type embryo proteome and phosphoproteome and can be explored via the Encyclopedia of Proteome Dynamics (EPD) – an open access, searchable online database.

## Introduction

Cellular diversity is based on the division of a cell into two unequal daughter cells; a process termed asymmetric cell division. Molecular mechanisms and key regulators of asymmetric cell division are conserved between vertebrates and invertebrates (for review see ref. 1). Thus, the nematode *C*. *elegans* with its largely invariant development has proven a valuable model to uncover pathways controlling asymmetric cell division. For example, studies into the first asymmetric cleavage of the *C*. *elegans* zygote led to the discovery of the polarity-regulating PAR (abnormal embryonic PARtitioning of cytoplasm) proteins, whose function is conserved in diverse animals (reviewed in ref. 2). In addition, a mechanistically conserved Wnt/β-catenin system resulting in unequal nuclear β-catenin levels in the two daughter cells has been described to act during several asymmetric divisions in *C*. *elegans* (reviewed in ref. 3). Finally, the transcription factor HAM-1 (HSN Abnormal Migration) and its target, the conserved kinase PIG-1 (PAR-1 (I)-like Gene), were both found to regulate asymmetric cell divisions resulting in an apoptotic daughter cell^4–6^. However, it is not known either whether HAM-1 and PIG-1 interact with the PAR proteins and/or the Wnt/β-catenin binary system, or how such potential interactions may be regulated.

Mutation of the transcription factor HAM-1 can result in survival of daughter cells destined for apoptotic cell death^5–7^. To the best of our knowledge, the kinase PIG-1 is the only known transcriptional target of HAM-1 involved in asymmetric cell divisions^8, 9^. Loss of *ham*-*1* and *pig*-*1* can transform the fate of daughter cells destined for apoptosis and result in them developing via the same neuronal fate as their surviving sister cells^7, 8^. Furthermore, mutation of either *ham*-*1*, or *pig*-*1*, alters the resulting sizes of daughter cells: thus, in *ham*-*1* mutants, the normally smaller apoptotic daughter cell is larger than the sister cell. In *pig*-*1* mutants and *pig*-*1*;*ham*-*1* double mutants daughter cell sizes are now similar in size^5, 8^. In relation to these cell size phenotypes, it is interesting that HAM-1 and PIG-1 were suggested previously to regulate myosin polarisation and the positioning of the mitotic spindle^9, 10^, which are processes known to influence daughter cell sizes (reviewed in refs 1, 11). However, the molecular mechanisms and proteins mediating these phenotypes that result from mutation of HAM-1 and PIG-1 are unknown.

PIG-1 is a member of the polarity-regulating PAR-1/Kin1/SAD-1 family of serine/threonine kinases^8^ and may play an important role in the regulation of asymmetric cell divisions. Localised phosphorylation events are known to contribute to the unequal segregation of cell fate determinants^1^ and PIG-1 expression was found at centrosomes^12^ and at the cortex of adjacent cells^13^. The expression of PIG-1 is ubiquitous in early embryos and becomes progressively more restricted in older embryos and young larvae, in which PIG-1 is expressed in dividing cells^8^. In addition to its role in asymmetric cell divisions, *C*. *elegans* PIG-1 was shown to control caspase-independent cell death and cell shedding, a process in which cells are eliminated by being actively expelled from their surrounding tissue^14, 15^. During cell shedding and asymmetric cell divisions, PIG-1 is controlled by the PAR-4-STRD-1-MOP-25 complex^12, 14^. Furthermore, PAR-4/LKB1 was shown to act via PIG-1 to regulate myosin accumulation in the *C*. *elegans* one-cell embryo^13^. In summary, PAR-4 appears to be genetically upstream of PIG-1, but the downstream effectors of PIG-1 are unknown.

The mammalian orthologues of HAM-1 and PIG-1 are connected to cell cycle regulation and cell death. The HAM-1 orthologue, Storkhead box (STOX) protein 1, is related to forkhead box (FOX) transcription factors^16^, which have a conserved role in cell cycle-regulated gene expression (for review see ref. 17). Furthermore, STOX1 itself was found to promote mitotic entry^18^ and the proliferation of inner ear epithelial cells^19^. The PIG-1 orthologue MELK (Maternal Embryonic Leucine zipper Kinase, also known as pEg3 kinase and MPK38) is predominantly expressed in proliferating cell populations and was reported to influence tumour growth and aggressiveness (for review see ref. 20). Putative MELK targets support a role in the regulation of cell cycle progression, cell proliferation and cell death^21–31^; however, the global effects of PIG-1/MELK kinase loss have not been investigated.

The *C*. *elegans* lineages that produce dopaminergic neurons provide a valuable system to explore the interplay between cell specification – a process that happens during every asymmetric cell division – and terminal cell differentiation – a mechanism that occurs only after the last mitotic division. *C*. *elegans* hermaphrodites possess 8 dopaminergic neurons: 2 CEPD (CEPhalic sensilla Dorsal), 2 CEPV (CEPhalic sensilla Ventral) and 2 ADE (Anterior DEirid) neurons in the head and 2 PDE (Posterior DEirid) neurons in the midbody. These dopaminergic neurons are derived from distinct lineages and their final differentiation is driven by a combination of dopaminergic ‘terminal selectors’, which are transcription factors controlling the expression of cell-specific features^32, 33^. However, the potential mechanistic connections between the HAM-1/PIG-1, PAR protein and Wnt/β-catenin specification pathways and subsequent dopaminergic neuron differentiation have not been characterised.

To identify factors influencing dopaminergic neuron specification and differentiation, we screened for *C*. *elegans* mutants exhibiting defective dopaminergic head neurons. We isolated a new allele of *ham*-*1* that causes an abnormal number of CEPD and ADE neurons and confirmed that the proposed downstream effector of *ham*-*1*, the kinase PIG-1, is also involved in the specification of CEPD and ADE neurons. We report epistasis between *ham*-*1* and *pig*-*1* and describe distinct genetic interactions with the apoptosis-defective mutant *ced*-*4* (*CEll Death abnormality*) in the lineages leading to dopaminergic head neurons. Using a global and phospho-specific mass spectroscopy-based proteomics approach, we screened for proteins affected by PIG-1 kinase loss. We found that *pig*-*1* mutation affects the abundance and phosphorylation status of key developmental proteins in the *C*. *elegans* embryo. In addition, we provide an in-depth characterisation of the phosphoproteome of *C*. *elegans* wild-type embryos. These results are accessible via the Encyclopaedia of Proteome Dynamics (EPD) − http://www.peptracker.com/epd/ − a searchable, open-access online database^34^ providing convenient tools for developmental biologists to explore and visualise these complex, large-scale proteomics datasets.

## Results

### *ham*-*1* mutation causes distinct defects in dopaminergic head neurons

**Figure 1:**
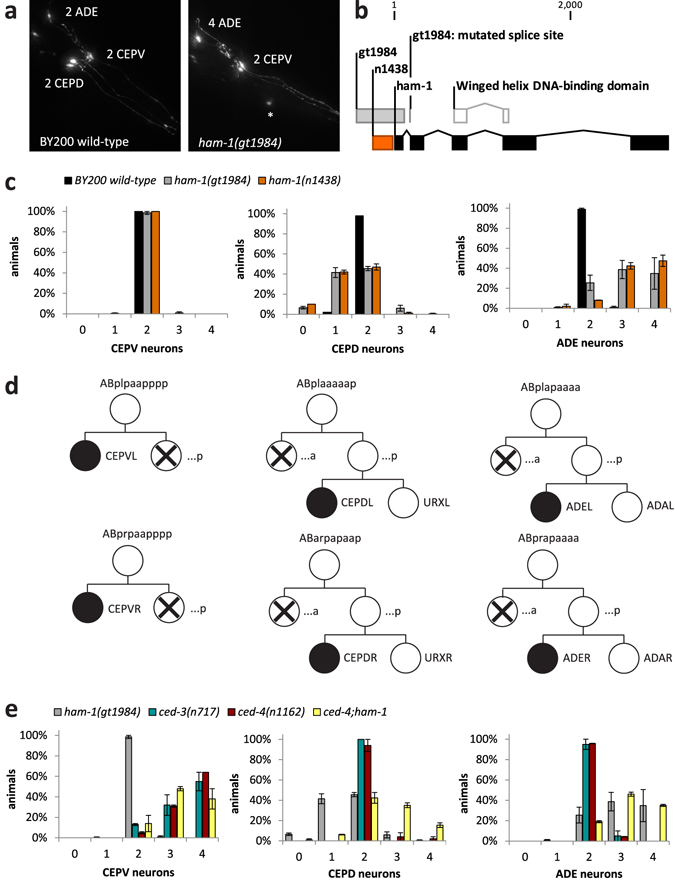
Distinct defects caused by *ham*-*1* mutation and genetic interactions with the apoptosis pathway in the lineages leading to *C*. *elegans* dopaminergic head neurons. (**a**) Dopaminergic head neurons in BY200 wild-type animals and in *ham*-*1*(*gt1984*) mutants. The dorsal cephalic sensilla (CEPD) neurons are located more posteriorly than the ventral cephalic sensilla (CEPV) neurons. A misplaced CEPD neuron is marked with an asterisk. (**b**) *ham*-*1* gene structure with winged helix box DNA-binding domain (in white) and positions of mutations in *ham*-*1*(*gt1984*) (in orange) and *ham*-*1*(*n1438*) (in grey). *gt1984* comprises a 554 base pair (bp) deletion spanning from the *ham*-*1* promoter into the first exon and also includes a splice site mutation at the start of the second exon. The 238 bp deletion of the *n1438* allele is within the *gt1984* deletion. (**c**) Number of dopaminergic head neurons in BY200 wild-type and *ham*-*1* mutant animals. Error bars = SEM of 2−4 biological replicates with 50 animals each. (**d**) Cartoon depicting the last divisions in the cell lineages that produce *C*. *elegans* dopaminergic head neurons (dopaminergic neurons are indicated with a solid black circle): left and right ventral cephalic sensilla neurons (CEPVL/R), left and right dorsal cephalic sensilla neurons (CEPDL/R) and left and right anterior deirids neurons (ADEL/R). Cells undergoing developmental apoptosis are marked with a cross. Daughter cells resulting from an anteroposterior division are labelled with ‘a’ and ‘p’, respectively, while daughter cells resulting from a left-right division are labelled with ‘l’ and ‘r’, respectively, starting from the ‘AB’ precursor cell. (**e**) Number of dopaminergic head neurons in the *ham*-*1* mutant, in the apoptosis pathway mutants *ced*-*3* and *ced*-*4* and in the *ced*-*4*;*ham*-*1* double mutant. Error bars = SEM of 2−4 biological replicates with 50 animals each.

To find genes that affect dopaminergic neuron development, a mutagenised population of the BY200 strain that allows for visualisation of *C*. *elegans* dopaminergic neurons (P*dat*-*1*::*gfp*)^35^ was screened for defective dopaminergic head neurons. One of the isolated mutants, *gt1984*, exhibited distinct phenotypes in dopaminergic head neurons: while the number of ventral cephalic sensilla (CEPV) neurons was unchanged, the number of dorsal cephalic sensilla (CEPD) neurons was decreased and the number of anterior deirids (ADE) neurons was increased (Fig. 1a,c). Based on the largely invariant cell lineages of *C*. *elegans* ^36, 37^, it can be derived that the respective ‘sister’ cells of CEPVL and CEPVR neurons die by apoptosis (Fig. 1d − left panels). In contrast, in the lineages of left and right CEPD and ADEL neurons it is an ‘aunt’ cell that becomes apoptotic (Fig. 1d − middle and right panels).

We mapped^38, 39^ *gt1984* to the *ham*-*1* (*HSN Abnormal Migration*) locus and found that the mutation comprises a deletion and a splice site mutation (Fig. 1b). Thus, *gt1984* is expected to result in a loss-of-function of HAM-1, an orthologue of the Storkhead box protein 1 (STOX1) transcription factor^6, 9^ containing a winged helix DNA binding motif (Fig. 1b). We verified that the same dopaminergic head neuron defects occur in a second *ham*-*1* mutant^4^ (Fig. 1c). While *ham*-*1* mutants were found to exhibit dopaminergic neuron defects^5, 40^, genetic interactions in these lineages have not been described. We conclude that *ham*-*1*(*gt1984*) constitutes a new loss-of-function allele that differentially affects cell fate in dopaminergic head neuron lineages.

Apoptosis in *C*. *elegans* development is controlled by the *cell death abnormality* genes *ced*-*3* and *ced*-*4*, which encode an executioner caspase and an Apaf1 (Apoptotic Peptidase-Activating Factor 1)-like molecule, respectively^41^. We tested if dopaminergic head neuron specification is affected by mutation of *ced*-*3* and *ced*-*4* alone. Blinded studies showed that the number of CEPV neurons - which possess an apoptotic sister cell – is increased in the *ced*-*3* and *ced*-*4* mutants; however, the number of CEPD and ADE neurons – which possess an apoptotic aunt cell – is unchanged (Fig. 1e). This observation is in line with studies showing that surviving cells in either the *ced*-*3* or *ced*-*4* mutants can adapt sister cell fates, but do not divide further to produce differentiated cells^5, 41, 42^. Moreover, we found that mutation of *ham*-*1* in the apoptosis-deficient background did not result in a further increase in CEPV neuron numbers (Fig. 1e – left panel). Therefore, the prevention of cell death appears to be sufficient to transform the normally apoptotic sisters into additional CEPV neurons.

Cell fate transformations caused by mutation of *ham*-*1* can be masked by cell death^5–7^. If this is true for the dopaminergic head neuron lineages, the lower number of CEPD neurons in the *ham*-*1* mutant is expected to be reversed in an apoptosis-deficient mutant background. Indeed, we found that in the *ced*-*4*;*ham*-*1* double mutant CEPD neuron numbers are either restored to wild-type levels, or are even higher (Fig. 1e – middle panel). However, the already increased number of ADE neurons in the *ham*-*1* mutant was not altered by additional mutation of *ced*-*4* (Fig. 1e – right panel). Hence, *ham*-*1* leads to cell fate transformations in both CEPD and ADE neurons, however in CEPD neurons the transformed cells appear to die via apoptosis.

### *pig*-*1* genetically interacts with *ham*-*1* to specify dopaminergic neurons

**Figure 2:**
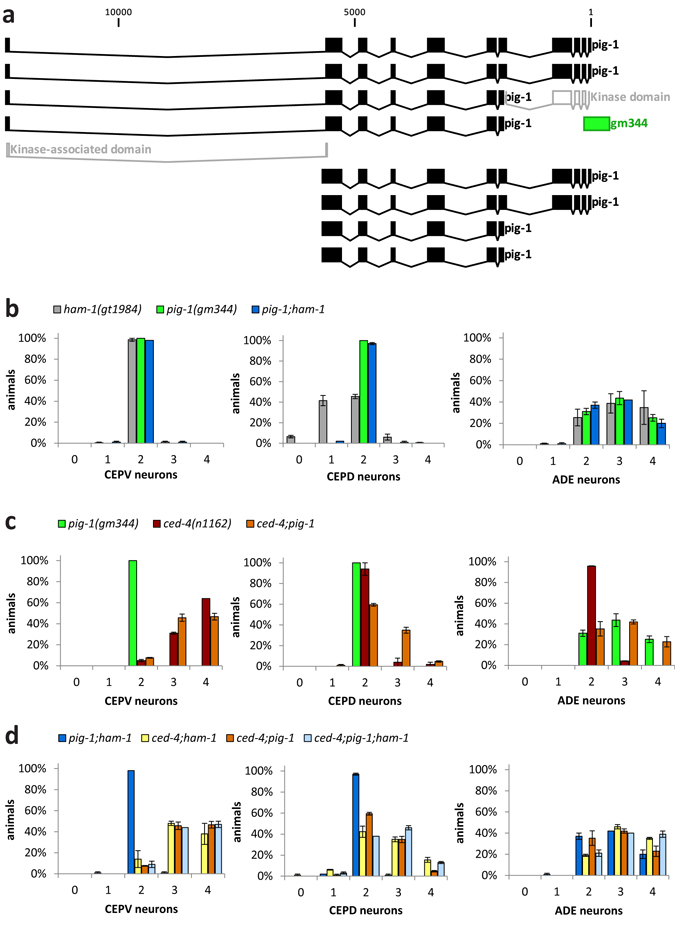
*pig*-*1* is epistatic to *ham*-*1* in the CEPD neuron lineage and genetically interacts with the apoptosis pathway. (**a**) *pig*-*1* gene structure with position of the predicted kinase and kinase-associated domains (both in white) and the *pig*-*1*(*gm344*) 562 bp deletion (in green). In *gm344*, 400 bp of the *pig*-*1* promoter and the first 132 bp of the kinase domain are deleted. (**b**) Number of dopaminergic head neurons in the *ham*-*1*, *pig*-*1* and *pig*-*1*;*ham*-*1* mutant. (**c**) Number of dopaminergic head neurons in the *pig*-*1*, *ced*-*4* and *ced-4;pig-1* mutant. (**d**) Number of dopaminergic head neurons in the double and triple mutants of *ham*-*1*, *pig*-*1* and *ced*-*4*. Error bars = SEM of 2–4 biological replicates with 50 animals each.

If PIG-1 is a general HAM-1 effector, *pig*-*1* and *ham*-*1* mutants are expected to show similar dopaminergic neuron defects. To determine *pig*-*1* mutant phenotypes, we used *pig*-*1*(*gm344*), which likely represents a null allele (Fig. 2a). We found that both *pig*-*1*(*gm344*) single mutants and *pig*-*1*;*ham*-*1* double mutants exhibited a wild-type number of CEPV neurons. Thus, CEPV neurons are not affected by loss of *ham*-*1* and *pig*-*1* (Fig. 2b – left panel). Furthermore, *pig*-*1* mutants show a higher number of ADE neurons, similar to *ham*-*1* mutants and *pig*-*1*;*ham*-*1* double mutants (Fig. 2b – right panel). Hence, *pig*-*1* and *ham*-*1* appear to repress ADE cell fate via a similar mechanism. However, in contrast with *ham*-*1* mutants, *pig*-*1* mutants did not display an decreased number of CEPD neurons (Fig. 2b – middle panel and ref. 8) and mutation of *pig*-*1* rescued the reduced number of CEPD neurons in the *ham*-*1* mutant (Fig. 2b – middle panel). Therefore, *pig*-*1* acts as a suppressor of neuronal fate in both CEPD and ADE lineages and is epistatic to *ham*-*1* in CEPD neurons.

We next tested genetic interactions between *pig*-*1* and the *ced*-*4* apoptosis gene in dopaminergic neuron lineages. We found that CEPD neuron numbers were higher in the *ced*-*4*;*pig*-*1* double mutant, even though *ced*-*4* and *pig*-*1* single mutants did not exhibit a phenotype (Fig. 2c – middle panel). This synergism is likely caused by the unmasking of a cell fate conversion in the apoptosis-deficient background, as is the case for *ham*-*1* and *ced*-*4* in CEPD neurons (Fig. 1e). In contrast, the higher number of CEPV neurons in the *ced*-*4* mutants was not further increased by mutation of *pig*-*1* (Fig. 2c – left panel). Also, the higher number of ADE neurons in the *pig*-*1* mutants was not further increased by mutation of *ced*-*4* (Fig. 2c – right panel). These results show that only one of the three lineages leading to dopaminergic head neurons shows a synergistic genetic relationship between *pig*-*1* and the apoptosis pathway as reported for other *C*. *elegans* lineages^8^. We further examined the phenotype of the *ced*-*4*;*pig*-*1*;*ham*-*1* triple mutant and found that in the ADE neuron lineage – in contrast to the CEPV and CEPD neuron lineages – mutation of *ced*-*4* did not lead to higher dopaminergic neuron numbers than in the *ham*-*1*;*pig*-*1* mutant background (Fig. 2d). In summary, we report varying genetic interactions between *ham*-*1*, *pig*-*1* and *ced*-*4* in the distinct dopaminergic head neuron lineages.

### Changes in protein abundance and signalling pathways in the *pig*-*1* mutant

**Figure 3:**
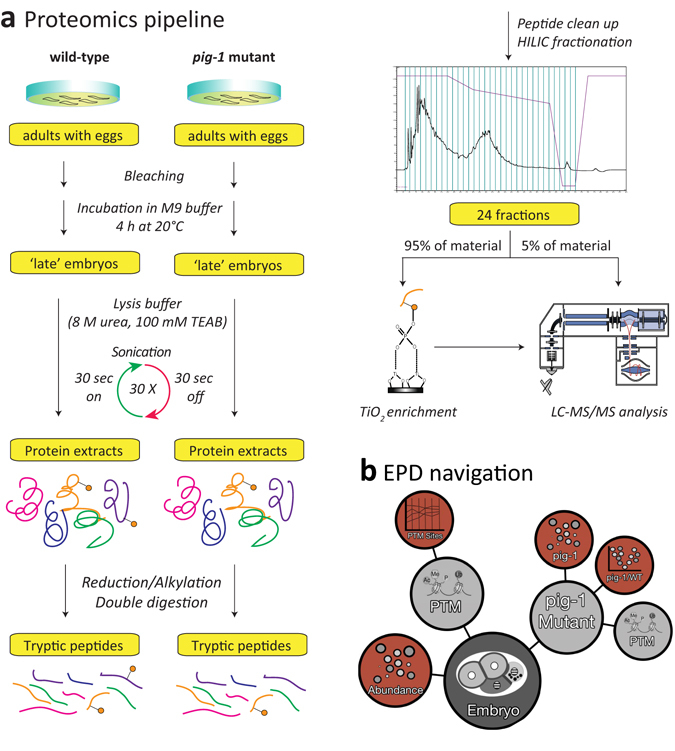
Mass spectrometry-based proteomics analysis and data deposition at the Encyclopedia of Proteome Dynamics (EPD). (**a**) Proteomics pipeline with a phosphopeptide enrichment step using titanium dioxide (TiO_2_) (for experimental details please refer to Materials and Methods). 95% of each peptide fraction was used to enrich for phosphopeptides using TiO_2_ affinity chromatography^43^ before being analysed by LC-MS/MS. In addition, 5% of the peptide fractions were directly measured with LC-MS/MS to assess global protein abundances. HILIC = hydrophilic interaction chromatography, TiO_2_ = titanium dioxide, LC-MS/MS = liquid chromatography tandem mass spectrometry. (**b**) Overview of protein abundance and post-translational modification (PTM) data from wild-type and *pig*-*1* mutant embryos available in the Encyclopedia of Proteome Dynamics (EPD).

To identify proteins and pathways that are affected by loss of the PIG-1 kinase, we turned to an unbiased, mass spectrometry-based proteomics approach (Fig. 3a). We aimed to determine PIG-1 targets in ‘late’ *C*. *elegans* embryos, corresponding to the developmental time point at which the last cell divisions of the dopaminergic head neuron lineage take place. Thus, we compared the proteomes and phosphoproteomes of wild-type and *pig*-*1* mutant embryos that were left to develop for 4 hours at 20 °C after isolation from a synchronised population of adult *C*. *elegans* hermaphrodites. Since PIG-1, which is an AMP-activated protein kinase-related protein, is likely to form part of a kinase cascade, we anticipate that both direct and indirect PIG-1 targets will be differentially phosphorylated in the *pig*-*1* mutant. In addition, proteins participating in pathways affected by *pig*-*1* mutation might also exhibit altered protein abundances. To facilitate interactive exploration of these large, complex datasets, the resulting protein abundance and protein phosphorylation data from the analysis of wild-type and *pig*-*1* mutant embryos described below, were incorporated into the Encyclopedia of Proteome Dynamics (EPD) database (Fig. 3b).

**Figure 4:**
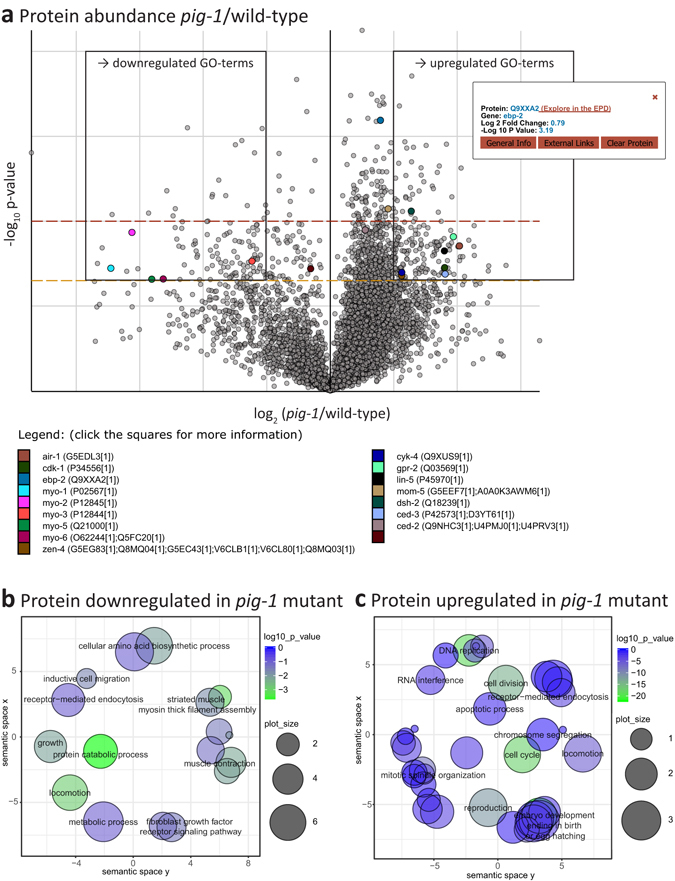
Global protein abundance changes in *pig*-*1* mutant embryos. (**a**) Volcano plot (in log_10_ vs. log_2_ scale) depicting protein abundance changes in the *pig*-*1* mutant embryo compared to wild-type embryos. The dashed orange and red lines indicate a p-value of 0.05 and 0.01 (1.3 and 2 in log_10_ scale), respectively. Boxed proteins were at least 2-fold down- or upregulated with a p-value of 0.05 and used for gene ontology term analysis. An interactive version of this volcano plot is available online via the EPD. To illustrate its functions, several proteins are highlighted and for one of them a tooltip with further available information is displayed. (**b** and **c**) Gene ontology term (GO) enrichment (biological process) of *pig*-*1*-variant proteins (boxed in the volcano plot). x and y-axis indicate semantic space used to group GO terms of related biological processes. Bubble sizes indicate the frequency of the GO term in the underlying *C*. *elegans* protein database (larger bubbles reflect more common terms) and bubble colour indicates statistical significance (the greener, the lower the p-value). (**b**) GO term analysis of proteins with at least 2-fold downregulated abundance in the *pig*-*1* mutant (Supplementary Table 5a). (**c**) GO term analysis of proteins with at least 2-fold upregulated abundance in the *pig*-*1* mutant (Supplementary Table 5b).

In the 5% of each peptide fraction that was used to measure protein abundance, we detected 51,414 unique peptides for wild-type and 48,690 unique peptides for the *pig*-*1* mutant, across the three biological replicates (Supplementary Table 1a). Peptides that were detected in at least two out of the three biological replicates were mapped to a total of 6,449 proteins for wild-type and 6,104 proteins for the *pig*-*1* mutant, respectively. Of these proteins, 184 were found to be >2-fold upregulated and 113 > 2-fold downregulated in *pig*-*1* mutant embryos, as compared with wild-type embryos, in at least two out of the three replicates (p-value < 0.05; false discovery rate (FDR) < 5% for both peptide and protein identification) (boxed in Fig. 4a) (Supplementary Table 2).

To investigate whether *pig*-*1* mutation alters the abundance of proteins associated with specific biological processes, we analysed the Gene ontology (GO) term annotations associated with both the down- and upregulated proteins detected (Supplementary Table 5a,b). GO terms associated with downregulated proteins encompass myosin-regulation (‘striated muscle myosin thick filament assembly’, ‘muscle contraction’), locomotion (‘locomotion’, ‘inductive cell migration’) and metabolism (‘protein catabolic process’, ‘growth’, ‘cellular amino acid biosynthetic process’ and ‘metabolic process’) (Fig. 4b). An increase in abundance was observed for proteins linked to cell cycle regulation (‘DNA replication’, ‘cell cycle’, ‘cell division’, ‘mitotic spindle organisation’), embryo development, apoptosis (Fig. 4c) and microtubule regulation (Supplementary Table 5b). We conclude that *pig*-*1* mutation leads to an overall decrease of proteins related to actomyosin processes, locomotion and metabolism and to an overall increase of proteins connected to the cell cycle, embryo development, apoptosis and microtubules.

**Figure 5:**
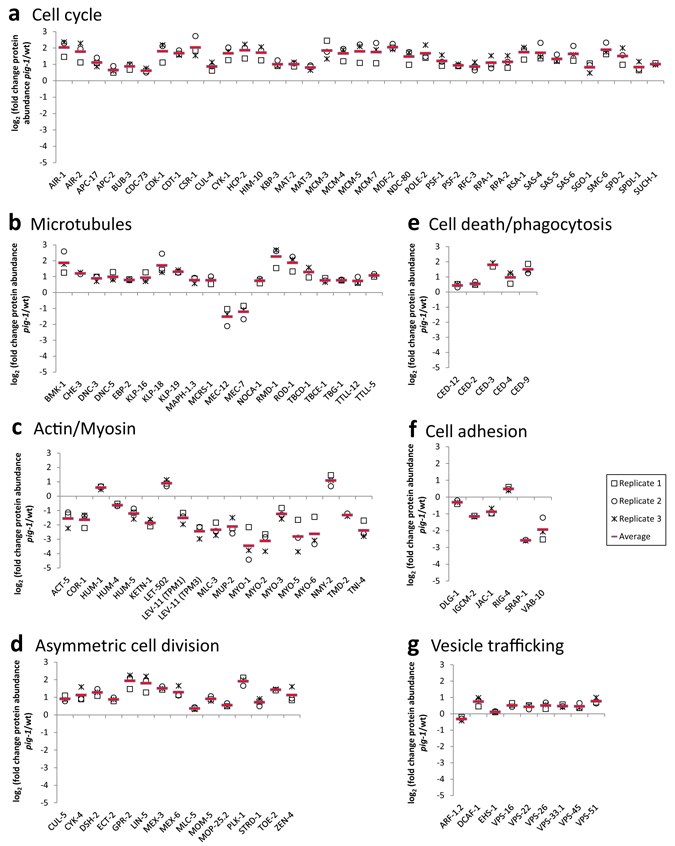
Abundance changes of key pathway proteins in *pig*-*1* mutant embryos. Proteins exhibiting a significantly different abundance (in log_2_ scale) in *pig*-*1* mutant embryos as compared to wild-type embryos, in at least two out of the three biological replicates (p-value < 0.05, two-tailed t-test; FDR < 5%) (replicate values are indicated in black and the calculated average over the replicates is indicated in red). Proteins are grouped according to their functional association with. (**a**) cell cycle regulation, (**b**) microtubule regulation, (**c**) actomyosin regulation, (**d**) asymmetric cell division, (**e**) cell death or phagocytosis, (**f**) cell adhesion, or (**g**) vesicle trafficking.

We next examined selected proteins within these highlighted biological processes showing altered protein abundances in *pig*-*1* mutant embryos. The selected proteins connected to either cell cycle regulation (Fig. 5a), or microtubule organisation (Fig. 5b), were more abundant in the *pig*-*1* mutant than in wild-type embryos. In contrast, selected proteins connected to actomyosin regulation were downregulated: For example, we detected a >5-fold lower protein abundance (note the logarithmic scale in Fig. 5) of MLC-3 (myosin light chain), MYO-1 (myosin heavy chain structural genes), MYO-2, MYO-4, MYO-5 and MYO-6 (Fig. 5c).

Next, we examined protein abundance changes in pathways previously connected to *pig*-*1* phenotypes. We found that proteins associated with asymmetric cell division, such as the centralspindlin complex-forming ZEN-4 (Zygotic epidermal ENclosure defective) and CYK-4 (CY to Kinesis defect) (reviewed in ref. 44), were more abundant in *pig*-*1* mutant embryos (Fig. 5d). Further examples include the >3.5-fold higher abundances detected for GPR-2 (G Protein Regulator) and LIN-5 (abnormal cell LINeage), the orthologues of *Drosophila* PINS (Partner of INScuteable) and NuMA (Nuclear Mitotic Apparatus protein), respectively. GPR-2 and LIN-5 mediate the formation of differently sized daughter cells via microtubule-cortex interactions in the *C*. *elegans* one-cell embryo (for review see ref. 1). We also observed that Wnt signalling components, such as the Wnt receptor MOM-5 (MOre of MS) and DSH-2 (DiSHevelled related), showed higher abundance in *pig*-*1* mutant embryos (Fig. 5d). Furthermore, as *pig*-*1* genetically interacts with the apoptosis pathway (Fig. 2c, refs 8, 14, 15) and as *pig*-*1* was suggested to promote the digestion of apoptotic cells^14^, we examined proteins related to cell death and corpse engulfment. We found that the apoptosis proteins CED-3 and CED-4 and the corpse engulfment proteins CED-2 and CED-12, all show increased abundance in *pig*-*1* mutant embryos (Fig. 5e). We also detected altered abundances of several cell adhesion proteins in *pig*-*1* mutant embryos (Fig. 5f). These data support the reported function of PIG-1 in cell detachment (‘shedding’) by interfering with the cell-surface expression of cell adhesion proteins in *C*. *elegans* caspase-deficient embryos^14^. PIG-1-mediated cell shedding was suggested to involve endocytosis^14^. Consistent with this proposal, we found that the vesicle trafficking protein ARF-1.2 (ADP-Ribosylation Factor-related), which is required for cell shedding^14^, was downregulated in *pig*-*1* mutants, while the abundance of other vesicle trafficking proteins was upregulated (Fig. 5g).

### Mutation of the PIG-1 kinase leads to phosphorylation changes in key development pathways

**Figure 6:**
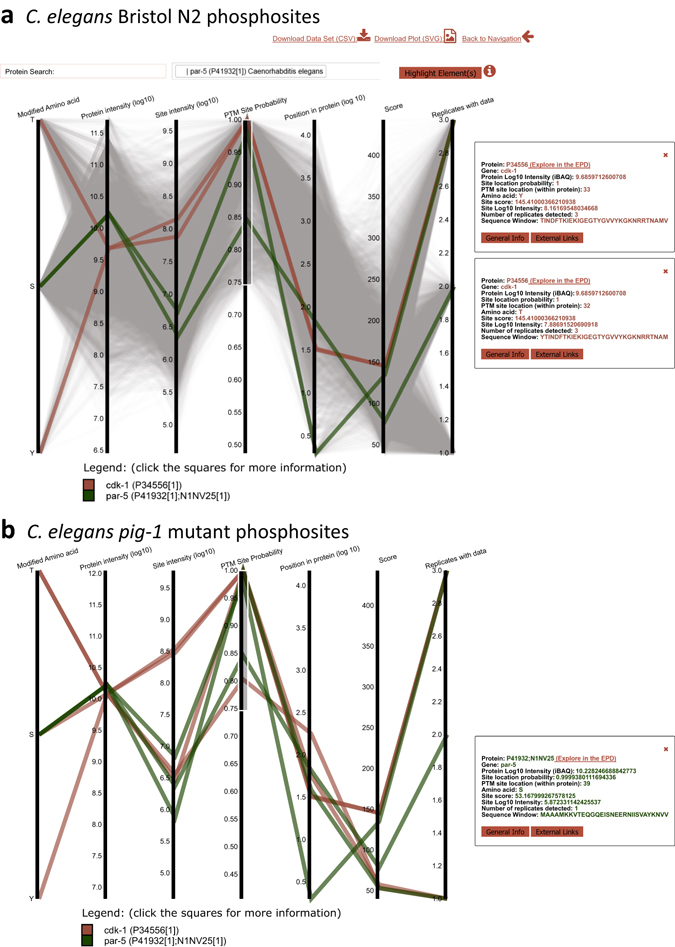
Phosphosites detected in wild-type and *pig*-*1* mutant embryos. (**a**) Detected protein post translational modification (PTM) phosphorylation sites in wild-type embryos. Each grey line represents a phosphorylation site and models its behaviour across multiple dimensions. The phosphorylation lines intersect the y-axes on the values that were detected for the specific site. The axis dimensions are ‘modified amino acid’ (threonine (T), serine (S) or tyrosine (Y)), ‘protein intensity’ (in log_10_ scale), ‘site intensity’ (in log_10_ scale), ‘PTM site probability’, ‘position in protein’ (in log_10_ scale), ‘score’ (of identification, the product of all peptide posterior error probabilities used for identification that is calculated in MaxQuant), and number of ‘replicates with data’. (**b**) Detected protein phosphorylation sites of two example proteins in *pig*-*1* mutant embryos - the grey lines for background phosphorylation were removed in this graph. Both of these plots are available online as interactive visualisations via the EPD (http://www.peptracker.com/epd/). Firstly, the axes can be filtered such that only elements contained within the user-defined boxes are shown: in the depicted graphs an example filter of ≥0.75 was applied for the ‘PTM site probability’ axis. Secondly, proteins can be searched and their phosphorylation sites can be highlighted in colour: Here, this was done for the same two example proteins and the search bar is shown on top of one of the graphs. Thirdly, selection of a phosphorylation line will lead to the display of a tooltip box containing further information: In the graphs shown, this was done for three examples of phosphorylation sites.

We hypothesised that both direct and indirect targets of the PIG-1 kinase may exhibit altered protein phosphorylation levels in *pig*-*1* mutant embryos. To test this hypothesis, we compared phosphopeptide abundances in *pig*-*1* mutant and wild-type embryos. Using a phosphopeptide enrichment step prior to liquid chromatography-mass spectroscopy analysis (Fig. 3a), we detected 7,247 phosphosites in wild-type embryos and 7,913 phosphosites in *pig*-*1* mutant embryos across the three biological replicates (FDR < 5%, localisation probability ≥75%) (Fig. 6) (Supplementary Table 1b). To facilitate the convenient exploration of this complex phosphoproteomics dataset, we created an interactive, searchable version of the data summarised in Fig. 6, which is available online via the EPD (http://www.peptracker.com/epd/). The plot features a custom visualisation tool that allows every phosphorylation site we identified to be selected and traced through a set of cognate metadata. We provide information about the abundance of each selected protein and its phosphosites, the amino acid position of each phosphosite in the protein, statistical evaluation of biological replicates, as well as linking to the raw mass spectrometry files. For wild-type embryos, these phosphosites correspond to 1,925 phosphorylated proteins (localisation probability ≥75%) (Supplementary Table 1b, Supplementary Table 3). To the best of our knowledge, this is the first dataset describing global protein phosphorylation in *C*. *elegans* embryos.

**Figure 7:**
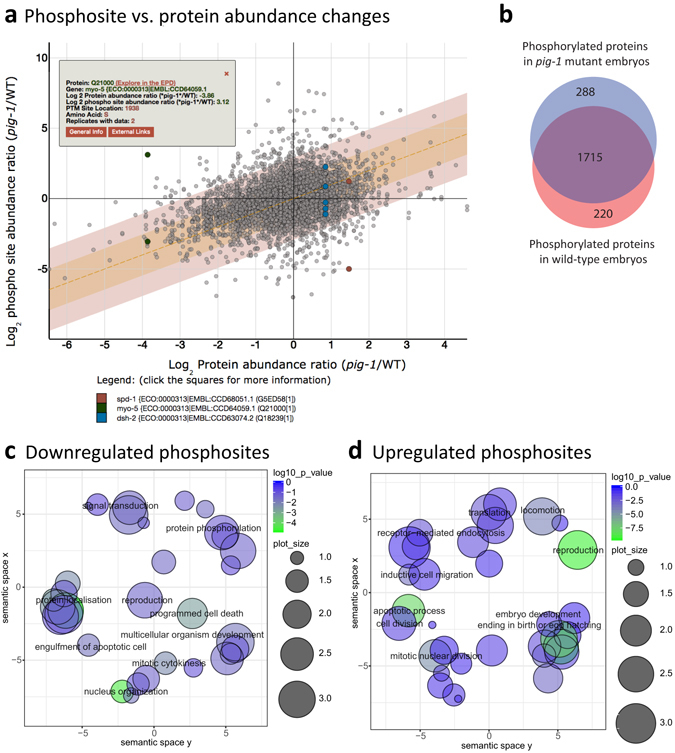
Global phosphoproteome changes in *pig*-*1* mutant embryos. (**a**) Phosphorylation site abundance ratio (*pig*-*1*/wild-type) compared to protein abundance ratio (*pig*-*1*/wild-type). The orange dotted line indicates where the phosphorylation site ratio is equal to the protein ratio. Points within the orange and red areas are within one and two standard deviations from equality, respectively. An interactive version of this plot is accessible online via the EPD. In this illustration, detected phosphorylation sites for three example proteins were marked and a tooltip containing further information is shown for one of these phosphorylation sites. (**b**) Number of phosphorylated proteins detected either in wild-type (red) or in *pig*-*1* mutant embryos (blue) or in both genetic backgrounds (purple overlap)(localisation probability >75%). (**c** and **d**) Gene ontology (GO) term enrichment (biological process) of *pig*-*1* variant phosphosites. x- and y-axis indicate semantic space used to group GO terms of related biological processes. Bubble sizes indicate the frequency of the GO term in the underlying *C*. *elegans* protein database (larger bubbles reflect more common terms) and bubble colour indicates statistical significance (the greener, the lower the p-value). (**c**) GO term analysis of downregulated phosphosites in the *pig*-*1* mutant (related to Supplementary Table 5c). Phosphosites downregulated in *pig*-*1* (outside of the 2 standard deviation range in (**a**)) were pooled with phosphosites that were only detected in wild-type embryos (red in (**b**)). (**d**) GO term analysis of phosphosites with upregulated abundance in the *pig*-*1* mutant (Supplementary Table 5d). Phosphosites upregulated in *pig*-*1* mutant embryos (outside of the 2 standard deviation range in (**a**)) were pooled with the phosphosites that were only detected in *pig*-*1* mutant embryos (blue in **b**)).

To help evaluate the significance of differences in the phosphoproteomes of *pig*-*1* mutant and wild-type embryos, we aimed to determine the stoichiometry of phosphorylation in relation to protein abundance. For this, we compared *pig*-*1*/wild-type phosphopeptide abundances to the corresponding *pig*-*1*/wild-type protein abundances (Fig. 7a). This showed that most proteins are located within two standard deviations of the diagonal of the plot, indicating that changes in phosphosite abundance tally with changes in protein abundance. Thus, these proteins are not significantly changing their stoichiometry of phosphorylation. However, proteins located more than two standard deviations from the mean were considered strong candidates for being differentially phosphorylated in the *pig*-*1* mutant, as compared with wild-type, embryos (Supplementary Table 4a). In addition to the differentially phosphorylated proteins, we identified 220 proteins that were exclusively phosphorylated in wild-type embryos and 288 proteins that were only phosphorylated in *pig*-*1* mutant embryos (Fig. 7b) (Supplementary Table 4b,c). The GO terms associated with proteins harbouring the respective up- and downregulated phosphosites were similar and connected to embryo development, the cell cycle and apoptosis (Fig. 7c,d). We note that this similarity in the biological processes connected with either increased, or decreased, levels of phosphorylation contrasts with our earlier finding that distinct processes were associated with the proteins showing either increased, or decreased abundance (Fig. 4b,c). In summary, we find that *pig*-*1* mutation leads to differential phosphorylation of multiple proteins associated with key developmental processes.

**Figure 8:**
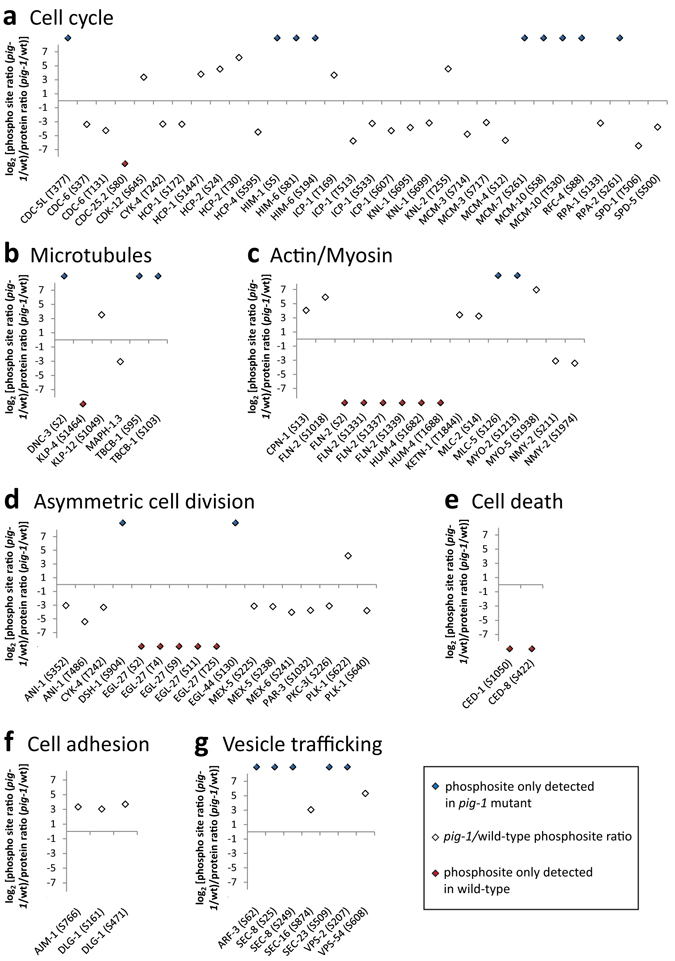
Phosphopeptide abundance changes of key pathway proteins in *pig*-*1* mutant embryos. Ratio of phosphopeptide abundance and protein abundance (in log_2_ scale) in *pig*-*1* mutants as compared to wild-type embryos (white diamonds). Phosphosites that were only detected in either *pig*-*1* mutants (blue diamonds), or wild-type embryos (red diamonds), are shown above and below the y-axis, respectively. Proteins are grouped according to their functional association with (**a**) cell cycle regulation, (**b**) microtubule regulation, (**c**) actomyosin regulation, (**d**) asymmetric cell division, (**e**) cell death or phagocytosis, (**f**) cell adhesion, or (**g**) vesicle trafficking.

Next, we examined the phosphorylation of selected proteins groups associated with changes in either protein abundance, or protein phosphorylation levels, in *pig*-*1* mutant embryos, as determined by GO term analysis. As seen in the phosphosite GO term analysis (Fig. 7e,f), *pig*-*1* mutation differentially affects phosphorylation of proteins mediating similar processes (Fig. 8). For example, phosphorylation of S80 in the cell division cycle protein CDC-25.2 was only detected in wild-type embryos, while phosphorylation of CDC-5L T377 was only detected in *pig*-*1* mutants (Fig. 8a). Furthermore, phosphorylation of S58 and T530 in the DNA replication protein MCM-10 [yeast MCM (licensing factor) related] is only detected in *pig*-*1* mutant embryos, while phosphorylation of ICP-1 (INCENP (inner centromere protein) homolog) is decreased at least 10-fold at 4 different sites (Fig. 8a) (note logarithmic scale in Fig. 8).

We found that *pig*-*1* mutation affects the phosphorylation of proteins previously associated with *pig*-*1* function. PIG-1 was shown to regulate the localisation of NMY-2 (Non-muscle MYosin)^9, 10, 13^ and NMY-2 itself affects the asymmetric distribution of several polarity proteins in the *C*. *elegans* one-cell embryo^1^. We found that in *pig*-*1* mutant embryos, NMY-2 S211 and S1974 are ~8-fold and ~10-fold less phosphorylated (Fig. 8c). In addition, PIG-1 was shown to control myosin accumulation in a pathway parallel to ANI-1 [ANIllin (actin-binding protein)]^13^ and we found that ANI-1 S352 is over 8-fold and ANI-1 T486 over 40-fold less phosphorylated in *pig*-*1* mutants (Fig. 8d). Furthermore, when investigating apoptotic corpse engulfment proteins, we found that CED-1 S1050 and CED-8 S411 are only phosphorylated in wild-type animals (Fig. 8e).

PIG-1 was suggested to affect the expression of cell adhesion proteins^14^ and we found that AJM-1 (Apical Junction Molecule) S766 and DLG-1 (*Drosophila* Discs LarGe homolog) S161 and S471 exhibit increased phosphorylation in *pig*-*1* mutant embryos (Fig. 8f). DLG-1 is a MAGUK (membrane-associated guanylate kinase) protein that mediates a microtubule-dependent pathway important for the segregation of cell fate determinants in *Drosophila* (reviewed in ref. 1). Furthermore, the inappropriate cell-surface expression of cell-adhesion molecules in *pig*-*1* mutants was proposed to be based on impaired endocytosis^14^, a process that appears to play an important role in asymmetric cell division^1^. We found increased phosphorylation for several endocytosis-associated proteins in *pig*-*1* mutant embryos (Fig. 8g).

Lastly, we found that *pig*-*1* mutation also affects the phosphorylation status of proteins connected to asymmetric cell division. PAR-3 (abnormal embryonic PARtitioning of cytoplasm) for example, the *C*. *elegans* orthologue of *Drosophila* Bazooka, exhibits decreased phosphorylation of S1032 (Fig. 8d). *pig*-*1* mutation also leads to altered phosphorylation of MEX-5 (Muscle EXcess), MEX-6 and PLK-1 (POLO Kinase) (Fig. 8d), proteins that segregate into the anterior daughter cell and help to establish the correct division of the *C*. *elegans* one-cell embryo (reviewed refs 1, 45). Furthermore, we found that phosphorylation of DSH-1 S904 is only detected in *pig*-*1* mutants (Fig. 8d). Dishevelled proteins are Wnt pathway components and normally enriched at the posterior part of an asymmetrically dividing cell^3^.

To provide the research community with convenient access to the results from this study, all the proteomics data describing protein and phosphopeptide abundance changes in the *pig*-*1* mutant embryos, as well as phosphopeptide detection in *C*. *elegans* wild-type embryos, have been incorporated in the Encyclopaedia of Proteome Dynamics (EPD) − http://www.peptracker.com/epd/. The EPD currently includes the largest collection of annotated, searchable mass spectrometry-based proteomics data from studies in *C*. *elegans*, linked with other extensive data sets from studies on human cells and model organisms. In addition, the corresponding raw mass spectrometry data files are freely available via the PRIDE repository, a partner in the ProteomXchange Consortium (http://proteomecentral.proteomexchange.org/).

## Discussion

We report that the transcription factor HAM-1 and the kinase PIG-1 have a role in the specification of *C*. *elegans* dopaminergic head neurons. *ham*-*1* and *pig*-*1* appear to be required for asymmetric cell divisions that result in an apoptotic daughter cell and either a neuron, or a neuronal precursor^5, 6, 8, 9^. In the case of dopaminergic head neurons, the last cell division in the CEPVL/R neuron lineages and the second last division for the CEPDL/R and ADEL/R neuron lineages, are the first ones to directly produce such an apoptotic daughter cell (Fig. 1d, http://wormweb.org/celllineage). We therefore consider it likely that *ham*-*1* and *pig*-*1* affect the asymmetric division of these neuroblasts. Genetic interactions in the CEPD lineage support that mutation of *ham*-*1* and *pig*-*1* might lead to sister-sister cell fate conversions, thereby turning a normally apoptotic aunt cell into a dopaminergic neuron precursor cell. When blocking cell death by mutation of the apoptosis pathway gene *ced*-*4*, these conversions are unmasked and result in higher neuron numbers. In addition, our experiments support that the genetic interaction between *ham*-*1* and *pig*-*1* depends on the exact neuronal lineage analysed. *pig*-*1* was implicated as a transcriptional target and HAM-1 effector in the Q.a neuroblast^9^. Consistent with this, we find an increased number of ADE neurons in *ham*-*1* and *pig*-*1* single and double mutants. In contrast, the reduced number of CEPD neurons observed in *ham*-*1* mutants is supressed by *pig*-*1*.

A key feature of this study was the use of a combination of quantitative, mass spectrometry-based proteomic and phosphoproteomic analyses to provide an in-depth, unbiased characterisation of the global changes that occur after genetic disruption of the PIG-1 kinase in *C*. *elegans* embryos. Direct targets of the PIG-1 kinase are expected to exhibit lower abundance of phosphorylated peptides in *pig*-*1* mutants, as compared with wild-type embryos. However, by analysing overall protein phosphopeptide and protein abundance changes, we were also able to measure indirect effects of *pig*-*1* inactivation. The extent of differential phosphorylation between wild-type and *pig*-*1* mutant embryos suggests that the majority of detected phosphorylation sites affected may not depend on direct phosphorylation by PIG-1 kinase. We note that this is the first study to analyse the effects of a kinase mutation on the global phosphoproteome in *C*. *elegans*.

In addition to the comparison of wild-type and *pig*-*1* mutant embryos described above, this study represents the first global phosphoproteome analysis reported for *C*. *elegans* embryos. We identify 5,051 phosphosites, corresponding to 1,639 phosphorylated proteins, in *C*. *elegans* wild-type embryos, which are detected in at least two out of the three biological replicates (FDR < 5%, localisation probability >75%) (Supplementary Table 1b). In addition, we detect 288 proteins that are only phosphorylated in *pig*-*1* mutant embryos (Supplementary Table 4c). There are only a limited number of phosphoproteome studies in *C*. *elegans*, reporting the identification of ~6,800^46^, ~6,500^47^ and ~3,500^48^ phosphorylation sites, respectively, in adult animals. However, our dataset is the first to identify phosphosites in the embryo and thus represents a valuable resource for the investigation of *C*. *elegans* development. Comparison with the *C*. *elegans* dataset from PhosphoPep^48^, a protein phosphorylation database for model organisms, showed that ~50% of the phosphosites we detect in embryos are novel (Supplementary Table 6).

We found that mutation of the PIG-1 kinase preferentially affected proteins associated with the cell cycle and asymmetric cell division, supporting the idea that PIG-1 might regulate the timing of cell cycle events in the neuroblast and thereby influence neuroblast polarity^8^. We note that cell division, cell polarity and differentiation are all tightly coordinated during animal development (reviewed in refs 49, 50). In addition, our results indicate that PIG-1 influences proteins regulating myosin distribution and spindle positioning. These processes are known to influence asymmetric cell divisions (for review see refs 1, 11) and might thus provide clues as to why *pig*-*1* mutants exhibit daughter cells of approximately equal size. Finally, microtubule-associated proteins are more abundant and differentially phosphorylated in *pig*-*1* mutants. During asymmetric cell divisions, microtubules maintain the established cell polarity by mediating interactions between centrosomes and the cell cortex^1, 11^. Therefore, the affected candidate proteins offer a promising starting point for further research.

The mammalian PIG-1 homologue, MELK, has also been implicated in cell cycle regulation^21, 27–30^. For example, MELK was shown to phosphorylate the mitosis-promoting phosphatase CDC25B that promotes transition to mitosis^21^. Since MELK overexpression blocks the transition to mitosis^21, 29, 51^, CDC25B phosphorylation by MELK is assumed to be inhibitory and mutation of the MELK kinase is therefore expected to stimulate cell division. Moreover, MELK overexpression has been associated with tumour growth and aggressiveness (for review see ref. 20).

In summary, the data presented here provide important new insights into the regulatory processes and signalling events involving PIG-1 that control asymmetric cell division during neuronal differentiation in *C*. *elegans*. In addition, this first detailed phosphoproteomic analysis of *C*. *elegans* embryos, together with the new interactive data visualisation tools we have developed, provide a valuable resource for developmental biologists studying both *C*. *elegans* and other species.

## Methods

### *C*. *elegans* strains and maintenance

TG2435 Bristol, a backcrossed version of BY200^35^, was used as the wild-type strain. TG4200 (*ham*-*1*(*gt1984*) *IV*;*vtIs1* V) was isolated following ethyl methanesulfonate (EMS)-mutagenesis and backcrossed six times. Worms were maintained at 20 °C on nematode growth medium (NGM) plates seeded with *E. coli* strain OP50.

### Strains

**BY200** *vtIs*[p*dat-1*::*gfp*;*rol-6*] V

**TG2435** *vtIs*[p*dat-1*::*gfp*;*rol-6*] V

**TG4200** *ham*-*1*(*gt1984*) *IV*;*vtIs1* V

**TG4201** *ham*-*1*(*n1438*) *IV*;*vtIs1 V*

**TG4202** *ced*-*3*(*n717*);*vtIs1 V*

**TG4203** *ced*-*4*(*n1162*);*vtIs1 V*

**TG4204** *ced*-*4*(*n1162*);*ham*-*1*(gt1984) *IV*;*vtIs1 V*

**TG4205** *pig*-*1*(*gm344*) *IV*;*vtIs1 V*

**TG4206** *pig*-*1*(*gm344*) *IV*; *ham*-*1*(*gt1984*); *vtIs1 V*

**TG4207** *ced*-*4*(*n1162*) *III*; *pig*-*1*(*gm344*) *IV*;*vtIs1 V*

**TG4208** *ced*-*4*(*n1162*) *III*; *pig*-*1*(*gm344*) *IV*; *ham*-*1*(*gt1984*); *vtIs1 V*

### Microscopy

For live imaging of neurons, L4 stage and young adult *C*. *elegans* hermaphrodites were placed in a drop of 20 mM levamisole on a slide containing an agar pad. A 1.5 mm cover slip was then placed on each slide and animals were imaged with a DeltaVision microscope. Quantification was performed blind with a Zeiss Axio Scope microscope.

### Mass spectrometry

#### Protein extraction and digest

For protein extraction, twelve 9 cm plates containing a synchronised population of *C*. *elegans* gravid hermaphrodites (ca. 40,000 animals) were washed to remove already laid eggs. The unlaid eggs were collected by bleaching and let develop for 4 hours at 20 °C before adding 500 μL of lysis buffer (8 M urea in 100 mM triethyl ammonium bicarbonate (TEAB) pH 8.5). The *C*. *elegans* embryos were then flash-frozen in liquid nitrogen and stored at −20 °C. The samples were ultrasonicated using a Bioruptor (30 cycles: 30 sec on, 30 sec off), reduced using tris(2-carboxyethyl)phosphine (TCEP) (25 mM) for 30 minutes at room temperature, then alkylated in the dark for 30 minutes using 2-iodoacetamide (50 mM). Total protein was quantified using the EZQ assay (Life Technologies). The lysates were diluted with 100 mM TEAB 4-fold for the first digestion with endoprotease Lys-C (Wako), then further diluted 2.5-fold before a second digestion with trypsin. Lys-C and trypsin were used at an enzyme to substrate ratio of 1:50 (w/w). The digests were carried out overnight at 37 °C, then stopped by acidification with trifluoroacetic acid (TFA) to a final concentration of 1% (v/v).

#### Peptide desalting and solid phase extraction

Prior to fractionation, the protein digests were desalted using C18 Sep-Pak Cartridges (Waters). Cartridges were first activated with Acetonitrile (ACN) and equilibrated with 50% ACN in water according to the manufacturer’s protocol. The samples were loaded and washed 4 times with 500 μL water containing 0.1% TFA. The peptides were eluted into a fresh Eppendorf tube with 800 μL 50% ACN and then dried *in vacuo*.

#### Off-line HILIC fractionation

Hydrophilic interaction chromatography (HILIC) was performed on a Dionex UltiMate 3000 (Thermo Scientific) using a similar protocol to the method described previously^52–54^. The dried peptides were redissolved in 80% ACN incorporating 0.1% TFA. The peptides were resolved on TSK-gel amide 80- column (TOSOH) using an inverted organic gradient of solvent A (water, 0.1% TFA) and solvent B (ACN, 0.1% TFA). 24 fractions were collected in 96-well deep well plates.

#### Phosphopeptide enrichment

Phosphopeptide enrichment was carried out on the HILIC fractions with titanium IMAC (immobilised metal affinity chromatography) beads (MagReSyn) using the original protocol by Larsen *et al*.^43^ on an automated bead processing robot (KingFisher, Thermo Fisher Scientific) as described in Tape *et al*.^55^. The enriched phosphopeptide fractions were dried, redissolved in 5% formic acid and analysed in data-dependent mode on Q Exactive Plus Orbitrap mass spectrometer (Thermo Fisher Scientific).

#### Liquid chromatography electrospray tandem mass spectrometry analysis (LC-ES-MS/MS)

5% of the material was analysed using a Q Exactive Plus Orbitrap mass spectrometer (Thermo Fisher Scientific) equipped with a Dionex ultra high-pressure liquid chromatography system (nano RSLC) without enrichment in order to quantify changes in protein expression levels. RP-LC was performed using a Dionex RSLC nano HPLC (Thermo Scientific). Peptides were injected onto a 75 μm × 2 cm PepMap-C18 pre-column and resolved on a 75 μM × 50 cm reverse phase-C18 EASY-Spray temperature-controlled integrated column-emitter (Thermo Fisher Scientific) using a four hour multistep gradient from 5% B to 35% B with a constant flow of 200 nL/min. The mobile phases were: 2% ACN incorporating 0.1% FA (Solvent A) and 80% ACN incorporating 0.1% FA (Solvent B). The spray was initiated by applying 2.5 kV to the EASY-Spray emitter and the data were acquired under the control of Xcalibur software in a data-dependent mode.

95% of the material was subject to phosphopeptide enrichment and analysed on a Q Exactive Plus using a two hour gradient.

#### Database searching and protein quantification

The raw mass spectrometry data were processed using the MaxQuant software suite (version 1.5.2.8). Proteins and peptides were identified using the UniProt *C*. *elegans* reference proteome database (Swiss-Prot and Trembl separately), using the Andromeda search engine^56, 57^ with the following search parameters: carbamidomethylation of cysteines was defined as a fixed modification, while methionine oxidation, acetylation of N-termini of proteins, conversion of glutamine to pyro-glutamate and phosphorylation on STY were set as variable modifications. The false discovery rate was set to 5% for proteins and peptides.

#### Mass spectrometry data analysis

The volcano plot depicting protein abundances was generated based on the label-free quantification intensity of proteins present in at least two of the three biological replicates in wild-type and mutant embryos. Statistical significance was determined with a two-tailed Student’s t-test with a randomisation setting fixed at 250 on the Perseus software platform^58^. For phosphorylation site analysis, data analysis was performed in Python using the scipy statistical package. Only phosphosites with a localisation site probability higher than 75% and a score higher than 40 were considered. Elements present in both wild-type and *pig*-*1* mutant embryos were added to the phosphosite comparison analysis, while elements present in only one of the genotypes were separated into two genotype-specific output files and visualisations. In the comparison analysis, the mutant over wild-type phosphosite abundance and protein abundance ratios were calculated and converted into log_2_ format for visualisation purposes. In the genotype-specific analysis, a log_10_ transformation was applied to the protein intensity, phosphorylation site intensity and the position of the post-translational modification within the protein to render the visualisation more user-friendly. The visualisation is presented in D3.js and the data is stored in Cassandra.

#### Data depository

Data generated in this study is accessible via the Encyclopaedia of Proteome Dynamics (EPD)^34^ (http://www.peptracker.com/epd/) for convenient exploring and display. In addition, the raw mass spectrometry files as well as the MaxQuant output have been deposited to the ProteomeXchange Consortium (http://proteomecentral.proteomexchange.org) via the PRIDE^59^ partner repository with the dataset identifier PXD005752.

#### Gene ontology term analysis

Gene ontology (GO)-term enrichment analysis was performed with the DAVID functional annotation tool (https://david.ncifcrf.gov/)^60, 61^. The complete list of *C*. *elegans* proteins detected in mass spectrometry-based proteomics was used as background and the GO term subcategories ‘GOTERM_BP_DIRECT’ ‘GOTERM_CC_DIRECT’ and ‘GOTERM_MF_DIRECT’ selected for analysis. The results were analysed with REVIGO^62^ and plotted using the provided R script to remove GO term redundancy. In REVIGO, p-values were provided and the *C*. *elegans* database and the SimRel semantic similarity measure were used for analysis. A ‘medium’ REVIGO list size was selected.

## Electronic supplementary material

Supplementary Table Legends

Supplementary Dataset 1

Supplementary Dataset 2

Supplementary Dataset 3

Supplementary Dataset 4

Supplementary Dataset 5

Supplementary Dataset 6
