# Supplementary material for: Comparative genetic, proteomic and phosphoproteomic analysis of C. elegans embryos with a focus on ham-1/STOX and pig-1/MELK in dopaminergic neuron development
Source: Sci Rep. 2017 Jun 28;7:4314. doi: 10.1038/s41598-017-04375-4 (PMC5489525; doi:10.1038/s41598-017-04375-4)
Supplement: Supplementary file 1 — Supplementary Table Legends [file 41598_2017_4375_MOESM1_ESM.doc]

# **SUPPLEMENTARY INFORMATION**

**Comparative genetic, proteomic and phosphoproteomic analysis of *C. elegans* embryos with a focus on *ham-1*/STOX and *pig-1*/MELK in dopaminergic neuron development**

Sarah-Lena Offenburger^1^, Dalila Bensaddek^1^, Alejandro Brenes Murillo^1^, Angus I. Lamond^1^ and Anton Gartner^1^*

^1^Centre for Gene Regulation and Expression, School of Life Sciences, University of Dundee, Dundee DD1 5EH, UK

*Correspondence to [a.gartner@dundee.ac.uk](mailto:a.gartner@dundee.ac.uk)

#

Supplementary Table 1: Number of detected peptides, phosphosites and phosphorylated proteins.

1. Number of peptides detected in each of the three biological replicates in wild-type and *pig-1* mutant embryos.
2. Number of detected phosphosites and phosphorylated proteins in wild-type and *pig-1* mutant embryos.

Supplementary Table 2: Protein abundance changes in *pig-1* mutant embryos.

Proteins with significantly different abundances in *pig-1* mutants compared to wild-type embryos in at least two out of three biological replicates (related to **Figure 4**). Proteins that are at least 2-fold upregulated are labelled in blue and proteins that are at least 2-fold downregulated are labelled in red.

Supplementary Table 3: Phosphosites detected in *C. elegans* wild-type embryos.

Phosphosites that were detected in *C. elegans* wild-type embryos (related to **Figure 6a**).

Supplementary Table 4: Phosphosites altered in *pig-1* mutant embryos.

1. Phosphosites showing different abundance (≥2 standard deviations from the mean, ) in *pig-1* mutants compared with wild-type embryos (localisation probability ≥ 75%) (**related to Figure 7a**).
2. Phosphosites that are detected in wild-type embryos but not in *pig-1* mutants (localisation probability ≥ 75%) (related to **Figure 7b**).
3. Phosphosites that were detected in *pig-1* mutant but not in wild-type embryos (localisation probability ≥ 75%) (related to **Figure 7b**).

Supplementary Table 5: Gene Ontology (GO) terms and associated proteins.

1. GO terms associated with proteins showing at least 2-fold downregulated abundance in *pig-1* mutants compared to wild-type embryos (related to **Figure 4b**).
2. GO terms associated with proteins showing at least 2-fold upregulated abundance in *pig-1* mutants compared to wild-type embryos (related to **Figure 4c**).
3. GO terms associated with proteins showing decreased phosphosite abundance *pig-1* mutants compared to wild-type embryos (related to **Figure 7c**).
4. GO terms associated with proteins showing increased phosphosite abundance in *pig-1* mutants compared to wild-type embryos (related to **Figure 7d**).

Supplementary Table 6: Phosphosite comparison.

1. Summary phosphosite comparison.
2. Mapped high confidence phosphosites.
3. Mapped lower confidence phosphosites.
